# Advances in Nanocomposites: Preparation, Characterization, Properties, and Applications

**DOI:** 10.3390/molecules29245924

**Published:** 2024-12-16

**Authors:** Reshma B Nambiar, Anand Babu Perumal, Emmanuel Rotimi Sadiku

**Affiliations:** 1 College of Animal Science, Zhejiang University, Hangzhou 310058, China; 2 College of Biosystems Engineering and Food Science, Zhejiang University, Hangzhou 310058, China; 3 Institute of NanoEngineering Research (INER) and Department of Chemical, Metallurgical and Materials Engineering, Tshwane University of Technology, Pretoria West Campus, Staatsartillerie Rd., Pretoria 0183, South Africa

Nanocomposites are a class of nanomaterials wherein one or more phases, of a nano-sized dimension (zero dimensions, one dimension, and two dimensions), are embedded in ceramic, metal, or polymer materials, etc. [1]. Nanoparticles, nanorods, nanospheres, nanofibers, nanocrystals, and carbon nanotubes (CNTs) are examples of the discrete units of nanocomposite materials [2,3,4]. Nanocomposites exhibit multifunctional properties, such as high surface-to-volume ratios, high mechanical strength, high electrical or thermal conductivity, good chemical resistance, desirable optical properties, decreased gas and water permeabilities, flame retardancy, redox reactivity, and catalytic activity [5,6,7,8,9,10,11,12]. Researchers have utilized nanocomposite materials for a broad range of applications [13,14,15,16,17,18,19,20,21]. In this Special Issue, of the nineteen manuscripts received, twelve articles were published and, among these, ten were original research articles and two were review articles, which focused on the preparation, characterization, and applications of nanocomposites, including biopolymers, thin films, etc.

AbdulRasheed-Adeleke et al. [Contribution 1] developed a thermoplastic starch (TPS) bionanocomposite films with lignin fillers from bamboo plants which have a reduced moisture absorption tendency and improved mechanical properties due to the good filler/matrix interfacial adhesion. The results show that the films produced would be suitable for application as packaging materials for food preservation. In another study, Folorunso et al. [Contribution 2] evaluated the fabrication of hybrids of polyvinyl alcohol (PVA), polypyrrole (PPy), and a reduced graphene-oxide nanocomposite. The study confirmed that the electrical conductivity of polymer composites depends greatly on the weight fraction of the fillers used. The study also indicated that the deployment of the optimal characterization/simulation tools for the prediction of the electrical conductivity of polymer composites is critical. Zhou et al. [Contribution 3] prepared biodegradable composites made up of Poly-(butylene adipate-co-terephthalate) (PBAT), thermoplastic starch, hydrophobically modified nanofibrillated cellulose (HMNC), and green surfactant (sucrose fatty acid ester) via the melt mixing and film-blowing process (PBATHMNC), which exhibited good mechanical and barrier characteristics. The *Agaricus* mushrooms preserved using these films retained their commodity value after 11 days of preservation. The developed PBAT-HMNC composites were proven to be promising nanocomposite materials for food packaging [22].

Other studies focused on the ecofriendly synthesis of bioactive glass nanofiber scaffolds, nanorod MAZ zeolite, and graphene quantum dots (GQDs). In one approach, a novel 3D hollow mesoporous bioactive glass nanofiber scaffold was synthesized with a template-assisted sol–gel method using a bacterial cellulose (BC) as a template and a nonionic triblock copolymer (P123) as a pore-directing agent, as well as ethyl orthosilicate (TEOS), calcium nitrate tetrahydrate (CN), and triethyl phosphate (TEP) as glass precursors. The scaffold had nanofiber-like morphology and an interconnected three-dimensional network structure with a large specific surface area and a large pore volume and exhibited excellent apatite-forming ability and cellular biocompatibility, which could be used in bone tissue regeneration [Contribution 4].

Zhang et al. [Contribution 5] reported a green route for synthesizing nanorod MAZ zeolite (MAZ-N) by using a low-cost and environmentally friendly choline chloride as a template. The MAZ-N samples had good crystallinity and uniform porous structures. The study suggested the potential application of MAZ-N zeolites as a supporting catalyst compounds in industrial processes. Nancy et al. [Contribution 6] developed a one-pot, facile, and ecofriendly synthesis approach for the fabrication of GQDs via the pulsed laser irradiation of an organic solvent (toluene) without any catalyst. It was observed that the GQDs obtained displayed fluorescence features and exhibited excellent optical limiting properties, and it was expected to have potential applications in optoelectronics and light-harvesting devices.

A review paper by Ahari et al. [Contribution 7] discusses the role of AuNPs (gold nanoparticles) in improving active food packaging. There is a global concern about food loss due to reduced shelf life, hence they suggested that the administration of nanotechnology-based methods, such as metal-based nanoparticles, can reduce the unresolved issues of the shortened shelf life of food and the associated environmental concerns about packaging, especially those of green synthetic-based AuNPs in comparison to the conventional chemistry-based methods. The developed AuNPs have a large surface-to-volume ratio, and diminished biohazards, aimed at increasing stability and the induction of antimicrobial or antioxidant properties. However, it is necessary to consider the hazards, including the migration of AuNPs for food packaging purposes. Another review examines the importance of Zn for obtaining multifunctional materials with interesting properties by following certain preparation strategies, such as choosing an appropriate synthesis route, the doping and co-doping of ZnO (Zinc oxide) films to achieve conductive oxide materials with *p*- or *n*-type conductivity, and finally adding the desired polymers to the oxide systems for piezoelectricity enhancement. The authors mainly followed the results of studies from the last ten years through chemical routes, especially via the sol–gel and hydrothermal synthesis routes. They concluded that ZnO can be very useful as a seed layer, for the good adherence of the main layer to the substrate, thereby generating nucleation sites for nanowire growth, which could be useful across multiple applications [Contribution 8]. A study on ZnO was also conducted by Xiao et al. [Contribution 9], who worked on the grain boundary structures of ZnO-based ceramics that were manipulated by the incorporation of polytetrafluoroethylene (PTFE) and metal oxides, through the cold sintering process (CSP), which reduced the grain size, increased the grain boundary resistance and the Schottky barrier height.

Bilal et al. [Contribution 10] prepared novel platforms for horseradish peroxidase immobilization by testing a variety of hybrid and single nanomaterials of various origins such as chitosan, magnetic nanoparticles, and selenium ions. The physicochemical characterization of the obtained systems proved successful enzyme deposition on all the presented materials with an efficiency above 70%. Of all the tested materials, the most favorable for immobilization was the above-mentioned chitosan-based hybrid material. The selenium additive increased the immobilization yield of the enzyme and improved enzyme stability. The obtained results confirm the applicability of these nanomaterials as useful platforms for enzyme immobilization in the contemplation of the structural stability of an enzyme and the high catalytic activity of fabricated biocatalysts. In another study, Jamal et al. [Contribution 11] prepared a bilayer electrospun fibers for skin tissue engineering applications with increased cell attachment and proliferation. Different ratios of PHBV-PLLA (70:30, 80:20, and 90:10 *w*/*w*) blends were electrospun on previously formed electrospun PHBV membranes to produce their bilayers. The membranes had random fibers with porous morphology and the fabricated bilayer showed high mechanical strength as well as biocompatibility with good cell proliferation and cell attachment, showing potential for skin substitute applications.

Malektaj et al. [Contribution 12] developed Ca–alginate–montmorillonite (MMT) nanocomposite gels, which were able to preserve their integrity in a wide range of pH values. The experimental data showed that the elastic moduli increase linearly with a concentration of MMT at all pH values under investigation due to the formation of physical bonds between the alginate chains and the MMT platelets. The study showed that the prepared gel could be used in wide range of applications such as tissue engineering, drug delivery, and food packaging. In conclusion, the research area of nanocomposites is growing very rapidly, and many applications in different sectors are very promising.
